# The Social and Ecological Integration of Captive-Raised Adolescent Male African Elephants (*Loxodonta africana*) into a Wild Population

**DOI:** 10.1371/journal.pone.0055933

**Published:** 2013-02-20

**Authors:** Kate Evans, Randall Moore, Stephen Harris

**Affiliations:** 1 School of Biological Sciences, University of Bristol, Bristol, United Kingdom; 2 Elephants for Africa, Maun, Botswana; 3 Elephant Back Safaris, Maun, Botswana; University of Western Ontario, Canada

## Abstract

**Background:**

A rapid rise in the number of captive African elephants (*Loxodonta africana*) used in the tourism industry in southern Africa and orphaned elephants in human care has led to concerns about their long-term management, particularly males. One solution is to release them into the wild at adolescence, when young males naturally leave their herd. However, this raises significant welfare concerns: little is known about how well released elephants integrate into wild populations and whether they pose a greater threat to humans than wild elephants. We document the release of three captive-raised adolescent male African elephants in the Okavango Delta, Botswana.

**Methodology/Principal Findings:**

Despite having been part of a herd of working elephants for at least eight years, the three males progressively integrated into the complex fission-fusion society of wild bull elephants. In the three years following release, they showed no tendency to be closer to human habitation, and there were no significant differences between wild and captive-raised adolescent males in the total number of social interactions, size of ranges and habitat use. However, the captive-raised elephants sparred less and vocalised more, and spent more time alone and in smaller social groups. Thereafter the released elephants continued to expand their ranges and interact with both mixed-sex herds and males. One male was shot by farmers 94 months after release, along with ten wild elephants, on a ranch outside the protected area.

**Conclusions/Significance:**

We show that captive-raised adolescent male elephants can integrate into a wild population. Long-term studies are required to determine the longevity, breeding success, and eventual fate of released male elephants, but we identified no significant short-term welfare problems for the released elephants or recipient population. Release of captive-raised mammals with complex social systems is a husbandry option that should be explored further.

## Introduction

Translocation has been used for more than 200 years [1] to reintroduce species to areas from which they have been extirpated, to alleviate overpopulation, restock [2]–[5] and remove problem animals [6]–[9], although this can generate controversy [3], [10]. While releases using wild-caught animals are more successful than those using captive-raised animals [2], both have proved effective for conserving at least some wild populations [11].

It is important to consider the welfare of the animals, not least because this can affect release success [3], [12], [13]. Adolescence is the ideal time to release captive-raised individuals as the greater behavioural flexibility and adaptability of adolescents increases survival rates [12], [14], [15]. However, since captive-raised animals may show disorientation and seek human company [3], [10], their release must be handled responsibly, with post-release monitoring using satellite GPS collars for animals likely to move large distances and ground observations to monitor the welfare of the released individuals, the recipient population and, in the case of potentially dangerous animals, humans near the release site.

Managing surplus and/or problem captive animals is problematic, particularly for charismatic species where slaughter is not publicly acceptable and sanctuary space limited [16]. African elephants (*Loxodonta africana*) are increasingly being used in the safari industry in Africa to carry tourists on game-viewing rides and/or for other interactions. Their long-term management, particularly young males and individuals which do not integrate into the herd, raises concerns. Releasing them into the wild is an option [17], although there are no scientific data on release success, and translocating elephants without regard for their social structure has led to problems in both Africa and Asia [5], [18]–[21].

The behaviour, movements and habitat use of three adolescent male elephants released into the Okavango Delta, Botswana were compared with wild elephants in the same area to test the hypothesis that captive-raised male elephants can be released into the wild. Our main concerns were their history of affiliation with humans and ability to integrate into wild bull society [22], and whether they used appropriate habitats [23]. We predicted that, compared to wild adolescent males, the released elephants would (i) spend more time close to humans; (ii) tend to be alone or in smaller groups; (iii) spend less time interacting socially; (iv) use significantly smaller areas; and (v) use different habitats.

## Methods

### Study Area

We conducted the study in the Okavango Delta, an inland wetland covering 15,000 km^2^ in north-west Botswana, southern Africa. It includes the Moremi Game Reserve and several wildlife management areas (protected areas set aside for wildlife conservation and associated activities) and is a RAMSAR (wetland of international importance) site. While people live on the edge of the Delta, there are few human settlements in the Delta and tourist lodges (semi-permanent camps) are sparsely distributed [24]. The boma (enclosure) that held the elephants prior to release was at S19.41483, E22.58421 (decimal degrees) adjacent to two lodges (Abu and Seba) in wildlife management area NG26, part of the seasonal swamps in the western Delta [25]. There were three seasons: rainy (November–March), flood (April–September) and dry (October, sometimes into November). Temperatures during the study ranged from 3–22°C in winter (flood season), 6–38°C in summer (dry and rainy seasons).

### Released Study Animals

The released males (Mafunyane, Seba and Thando) were part of the Abu herd, used in the safari industry by Elephant Back Safaris (Pty) Ltd. They were cull orphans from Kruger National Park (Table 1), trained to obey verbal commands and carry a handler but never fitted with a saddle and ridden by tourists. They walked daily with the Abu herd when on safari and feeding in the bush, and were held in a boma at night. So they were familiar with the area around the boma and interacted with wild elephants, mainly vocally as wild elephants were discouraged from staying close to the herd.

**Table 1:** Details of the three male elephants released in Botswana and collaring dates.

Elephant	Estimated yearof birth	Year of cull and move to Botswana	Date of release	Estimated age (years) at release	Dates collared and type of collar
Mafunyane	1988	1989	1 Feb 2002	14	31 Jan 2002 fitted with DM 200 Imarsat Unite satellite collar programmed to record GPS locations every 12 hours; replacement Globaltrack AWTSM2000E collars programmed to record GPS locations every 8 hours fitted on 17 Nov 2002, 4 Feb 2004 and 9 Nov 2004
Seba	1994	1995	10 Feb 2003	9	8 Feb 2003 fitted with Globaltrack AWTSM2000E satellite collar programmed to record GPS locations every eight hours;recollared on 1 Apr 2004 and 10 Nov 2004 with same model and recording frequency
Thando	1987	1989	10 Feb 2003	16	8 Feb 2003 fitted with Globaltrack AWTSM2000E satellite collar programmed to record GPS locations every eight hours; recollared on 28 June 2004 with same model and recording frequency

All three were orphans from culls in Kruger National Park, South Africa and originally held at a different location in Botswana, although still part of the same herd of working elephants. The herd was moved to the release site in 1995.

The three males were released during adolescence, when male elephants reach puberty and become increasingly independent. Most male elephants are still in their natal herds during early adolescence (10–15 years), whereas most have dispersed by late adolescence (16–20 years) [26], [27]. While we estimated that Seba was about nine years old at release, he had been showing increasing signs of independence.

At the time of the releases we were not concerned about genetic maladaptions to local environmental conditions [28] or potential genetic dilution effects on the resident population [29] since the elephants in northern Botswana were not considered genetically distinct from those in Kruger [30]. Although a subsequent study found considerable variation in mitochondrial DNA [31], three individuals are unlikely to have an effect on the gene pool. To minimise the risk of introducing novel diseases [32], the Abu herd was under veterinary supervision and the released elephants showed no clinical signs of disease. Before release, a veterinarian tested them for *Mycobacterium tuberculosis*, as required by Botswana’s Department of Wildlife and National Parks (DWNP).

We used different release procedures. On 31 January 2002, Mafunyane was not returned to the boma with the rest of the Abu herd. He was fitted with a satellite collar, left chained in the bush about a kilometre away, and released the following morning. In January 2003 Seba and Thando were separated from the Abu herd and held at night in a separate boma 2.7 km away, and so were in vocal but not physical contact with the rest of the herd. For the next month they were taken to feed by themselves during the day, collared on 8 February 2003 and two days later walked 5 km south of their boma to feed for the day. At dusk their chains were removed and the elephant handlers departed.

We used two types of satellite collars (Africa Wildlife Tracking cc, Rietondale, Pretoria, South Africa; Table 1); both recorded to 10 m spatial accuracy and locations were downloaded daily from a website (http://mstrackweb8.skygistics.com). The original collars were fitted without anaesthesia; thereafter the released males were sedated to fit replacement collars as described below for the wild males. The released males were monitored intensively until February 2005; GPS data were collected thereafter, and behavioural data whenever they were accessible from the release site. Seba was shot in December 2010 (see Discussion), Thando’s collar removed on 19 September 2011 and Mafunyane’s on 14 October 2011.

Prior to the releases, photographs of the elephants were sent to all the lodges in the Delta and staff were asked not to approach the elephants but to report any sightings or incidents. Staff were also notified if the released elephants were moving towards their lodge or were within 5 km of it. To dissuade the released males from trying to rejoin the Abu herd, during the initial period of release they were located in the morning and evening and the elephant handlers or lodge staff chased them away if they were near or approaching the boma, or Abu or Seba lodges.

### Wild Study Animals

To obtain comparative data on wild elephants, on 16 and 17 February 2002 five adolescent males (Abu 2, Abu 3, Abu 4, Abu 5 and Abu 6) were randomly selected from female herds within 10 km of the boma and anaesthetised from a helicopter using gun-propelled syringes containing 12 mg of etorphine hydrochloride (M99) and 5000 international units of hilaze. After the anaesthetic took effect, the helicopter landed and the elephant was pushed onto his side if in a prostrate position, the collar fitted, and 25 mg of diprenorphine (M50/50) administered. The elephants were never sedated for more than 20 minutes and their recovery was monitored from the air by helicopter or fixed-wing aircraft until they moved away.

The wild males were originally fitted with DM 200 Imarsat Unite satellite collars (Africa Wildlife Tracking cc) programmed to record GPS locations every 12 hours. All five were recollared on 13 and 14 March 2003 with Globaltrack AWTSM2000E satellite collars (Africa Wildlife Tracking cc) programmed to record GPS locations every eight hours. Due to further technical problems, Abu 2 was recollared on 28 June 2004, Abu 4 on 7 December 2003, Abu 5 on 6 October 2003 and Abu 6 on 1 April 2004. The collars were removed from Abu 3 and 4 on 9 November 2004 and from Abu 2 and 6 on 5 November 2005. The collar on Abu 5 failed and so we could not relocate him.

The University of Bristol’s ethical review process and the DWNP approved the darting and handling procedures, which conformed to the American Society of Mammalogists’ guidelines [33]. Experienced wildlife veterinarians approved by the DWNP performed the anaesthesia. We made every effort to minimize suffering and recorded no adverse effects of anaesthesia or collaring.

### Data Collection

Both types of collar included a VHF transmitter. The released males were located from the ground with a Telonics TR-4 receiver (Telonics Inc., Mesa, Arizona, USA) on average five times per rainy season, nine times per flood season, and five times per dry season. Released and wild collared males were also tracked bimonthly from the air using a Piper J−3 Cub fixed-wing aircraft (1946 Model, 100 HP). H-aerials (African Wildlife Tracking cc) were attached to each wing at 45 degrees to the ground and linked to a Telonics TR-4 receiver through a switch box (African Wildlife Tracking cc). Once the elephants were located, they were circled to collect data on social grouping (Table 2), number and sex of nearby elephants, and distance from the nearest neighbour. This did not seem to disturb the elephants unduly, although solitary elephants tended to move into cover.

**Table 2:** Social groupings used in the analyses.

Code	Social grouping
**1**	Alone: no other elephants within 500 m
**2**	In a group of 1–5 males within 500 m of each other
**3**	In a group of >5 males within 500 m of each other
**4**	In a mixed herd: adult males and females within 500 m of each other
**5**	Within 500 m of the Abu herd

From February 2002 to February 2005, 30-minute focal observation periods were used to collect behavioural data for the released and wild collared males (Table 3). In addition, five set and some random routes were driven along dirt tracks traversing all the main habitats in the release area during the morning (05.30–11.59) and afternoon (12.00–19.00) to locate uncollared wild male elephants. These were allocated to one of five age classes (10–15, 16–20, 21–25, 26–35 and ≥36 years) using a combination of tusk size [34], measurement of footprints [35], [36], estimation of shoulder height [36]–[38] and physical characteristics such as tusk girth and head shape [26]. They were provisionally identified in the field from ear tears and other features, and an animal not known to have been sampled that season was selected for a 30-minute focal period. Later, identification was verified using film and digital photographic identification files for 417 males, 34 females and 15 calves. If the same animal had been sampled more than once per season, or more than one elephant had been sampled from the same social group per season, one focal period was selected at random for analysis.

**Table 3:** Activities recorded during half-hour focal observations; adapted from [64].

Code	Activity	Description
**1**	Sleeping	Standing in one place with eyes closed for longer than one minute while not feeding
**2**	Feeding	Chewing or using the trunk to manipulate food items
**3**	Drinking	Intake of water
**4**	Social behaviours	Focal elephant interacting with at least one other elephant
**4.1**	Greeting	Raises trunk to mouth of another elephant
**4.1.1**		Another elephant greets focal elephant
**4.2/4.3**	Sparing/playing	Head to head contact and pushing between two or more elephants
**4.4**	Pushing from behind	Using tusks or resting trunk over back of the other elephant and pushing
**4.4.1**		Focal elephant is pushed from behind
**4.5**	Displaying	Destruction of vegetation without eating, crashing through vegetation, headshaking
**4.5.1**		Another elephant is displaying
**4.6**	Head over back	Standing or walking with head and/or trunk resting on back of another elephant
**4.6.1**		Another elephant with head and or trunk on back of the focal elephant
**5**	Mud bathing/dusting	Collection of dust or mud with trunk and then throwing it over themselves
**6**	Walking	Moving purposefully at a steady pace
**7**	Walking while feeding	Moving at a steady pace while chewing or manipulating food items
**8**	Standing	Standing in one place with eyes open for longer than one minute while not feeding
**9**	Vocalising	
**9.1**		Vocalisation by focal individual
**9.2**		Vocalisation by known other
**10**	Running	Moving at pace, generally when alarmed
**11**	Other	Focal elephant does another activity e.g. pushes over tree to eat
**11.1**		Another elephant does another activity

The focal individual’s social grouping (Table 2) and, where possible, identity of any other elephants, were recorded at the start of each focal period. The activity, habitat (see below), location (measured with Garmin GPS III plus, Garmin International Inc, Olathe, Kansas City, USA), and identification of and distance to the nearest neighbour were recorded every five minutes. Unusual behaviours outside these observation points, such as greeting and sniffing, were recorded, as were the length and rate of all audible vocalisations and caller identity, determined by audible (direction, intensity) and visual (ear flapping, listening, opening mouth) cues. Activities were quantified as the rate per 30 minutes; nearest-neighbour distance was averaged across the 30 minutes.

Wherever possible, the vehicle was parked some distance from the focal elephants. However, if they appeared disturbed (looked at the vehicle too frequently, tried to approach or move away), the vehicle was moved and a new focal period started if the elephant(s) resumed normal activity.

### Analysis of Social Behaviour

Released and wild collared males were compared for differences in distance to human habitation and social groupings, and released and wild (collared and uncollared) adolescent males were compared for differences in nearest neighbours, social interactions and social behaviours. Only samples when the focal animal was with one or more elephants were used to analyse social interactions; focal data when the elephant was alone were used when analysing rates of vocalisation. If the released males were seen three or more times in a day, a random sample was selected from both the morning and afternoon.

A GLM with Poisson distribution was used to test for differences in social groupings, with sightings with herds and the Abu herd combined (Table 2, Codes 4 and 5), and associations with males of different ages. χ^2^ tests were used to determine whether the social groupings of each released male differed from wild adolescent males. Kruskal-Wallis tests were used to compare social groupings of Mafunyane one, two and three years post release, Mann-Whitney tests to compare the social groupings of Seba and Thando.

Mann-Whitney tests were used to compare median nearest neighbour distances for the released and wild adolescent males and nested GLMs with Poisson distribution to compare differences in: the rate of all social interactions (sparring, greeting, other tactile behaviours); sparring and greeting, the two main social activities, separately; and vocalisations between the released and wild adolescent males, and amongst the released males, to see if any individual was influencing the overall vocalisation rate. The data were analysed using S-PLUS version 6.1 (Insightful Corporation, Seattle, Washington, USA). *F* values are given for data that were over dispersed, χ^2^ where they were not [39]. Two sample *t*-tests and Mann-Whitney tests were used as appropriate. Proportional data were log-ratio transformed [40] and compared using 1 or 2 sample *t*-tests, paired *t-*tests or one-way ANOVAs.

### Analysis of Ranging Behaviour and Habitat Use

Distance to the nearest human habitation was calculated using ArcGIS (Release 10, Environmental Systems Research Institute, Redlands, California, USA). The GPS points of all villages and lodges in Ngamiland, Botswana (Services for GeoInformation, Maun, Botswana) were included and analysed by fitting linear mixed models with maximum likelihood methods to log-transformed data using the Ime4 package in R version 2.5.1 (the R Foundation for Statistical Computing). Nested models were compared using the change in deviance on removal of a term and Akaike’s Information Criterion and simplified by removing non-significant terms to identify the most parsimonious model [41].

Home range size and area used by the released and collared wild adolescent males were calculated using the Animal Movement SA 2.0 extension in ArcView GIS version 3.2. 100% minimum convex polygons (100% MCPs) and 95% and 70% kernels were calculated for each collared elephant in the rainy and flood seasons and differences between the released and wild males were compared using *t*-tests. Patterns of habitat use were quantified using compositional analysis based on log-ratio transformed proportions [40] using Compos Analysis version 5.0 [42]. Because the number of habitats analysed must not exceed the number of individuals [43], the 47 habitat types [44] were combined into four groups: grassland/floodplain; mopane woodland (dominated by *Colophospermum mopane*); ‘other’ woodland (dominated by *Acacia* spp., *Combretum* spp. and *Terminalia sericea*); and island vegetation (dominated by *Hyphaene petersiana* and/or *Phoenix reclinata*).

Patterns of habitat use were compared at two levels: (i) the composition of individual areas used versus the composition of the total available habitat; and (ii) the proportional use of habitats, i.e. the proportion of an elephant’s GPS locations within each habitat versus the composition of the 100% MCPs. The area of available habitat within the Delta and within individual 100% MCPs was calculated using ArcView GIS version 3.2 and an existing habitat map [44]. In both analyses, habitats were ranked by preference when a lambda test (λ) showed that habitat use deviated significantly from random. To compare habitat use between released and wild males, the data were log-ratio transformed [40] and compared using a GLM. Analyses were conducted using MINITAB version 14 (MINITAB Inc., Pennsylvania, USA), the underlying assumptions were met for all tests [45], and the results considered significant where α = 0.05.

## Results

The released elephants were sighted 920 times, 760 of which were independent and used in the analyses. The collared wild elephants were sighted 434 times, all of which were independent, and uncollared wild males 4072 times. For the released males, 618 of 645 focal samples were independent: for 330 (53%) they were with wild elephants. Of 313 focal samples from wild adolescent males, 97 were independent. For 92 (95%) of these the focal male was with other elephants; on 16 occasions this was one or more of the released males.

Prediction (i), that the released males would spend more time close to humans, was not supported. A mixed-effects model with a common subject slope but different intercepts had a lower AIC than a model with varying slopes and intercepts (AIC 35175 vs 35176); the former was not a significantly poorer fit to the data (Δdeviance_5_ = 1.4148, P = 0.2343). When all human habitation was considered, the released males were significantly closer than the wild collared males (Δdeviance_4_ = 8.7562, P = 0.0031; AIC for model without x = 35182 vs 35175 with this term). However, when the two lodges associated with the elephants’ boma were excluded (Table 4), a mixed-effects model with a common subject slope but different intercepts had a lower AIC than one with varying slopes and intercepts (AIC 29601 vs 29603) and the former was not a significantly poorer fit to the data (Δdeviance_5_ = 0.0053, P = 0.9422). With Abu and Seba lodges excluded, the released males were not significantly closer to human habitation than the wild males (Δdeviance_4_ = 1.3215, P = 0.2503; AIC for model without x = 29600 vs 29601 with this term). From 2006–2011, the released males progressively moved further from their release site (Table 4).

**Table 4:** Mean distance (m±SE) of collared elephants from human habitation.

Elephants	Years	Mean distance to human habitation (all lodges andvillages)	Mean distance to human habitation excluding Abuand Seba lodges	No. of GPS fixes
**Mafunyane**	2002–2005	1730±33	12,504±45	3310
	2006–2011	7396±105	11,901±47	2378
**Seba**	2003–2005	3950±81	7867±132	1096
	2006–2010	11,346±232	12,088±217	986
**Thando**	2003–2005	3743±71	8527±114	1344
	2006–2011	4364±62	7026±102	1944
**All released males**	2002–2005	2624±33	10,343±52	5750
	2006–2011	7021±76	10,776±76	5308
**All wild collared males**	2002–2005	6656±38	7434±41	9979

**Figure 1:**
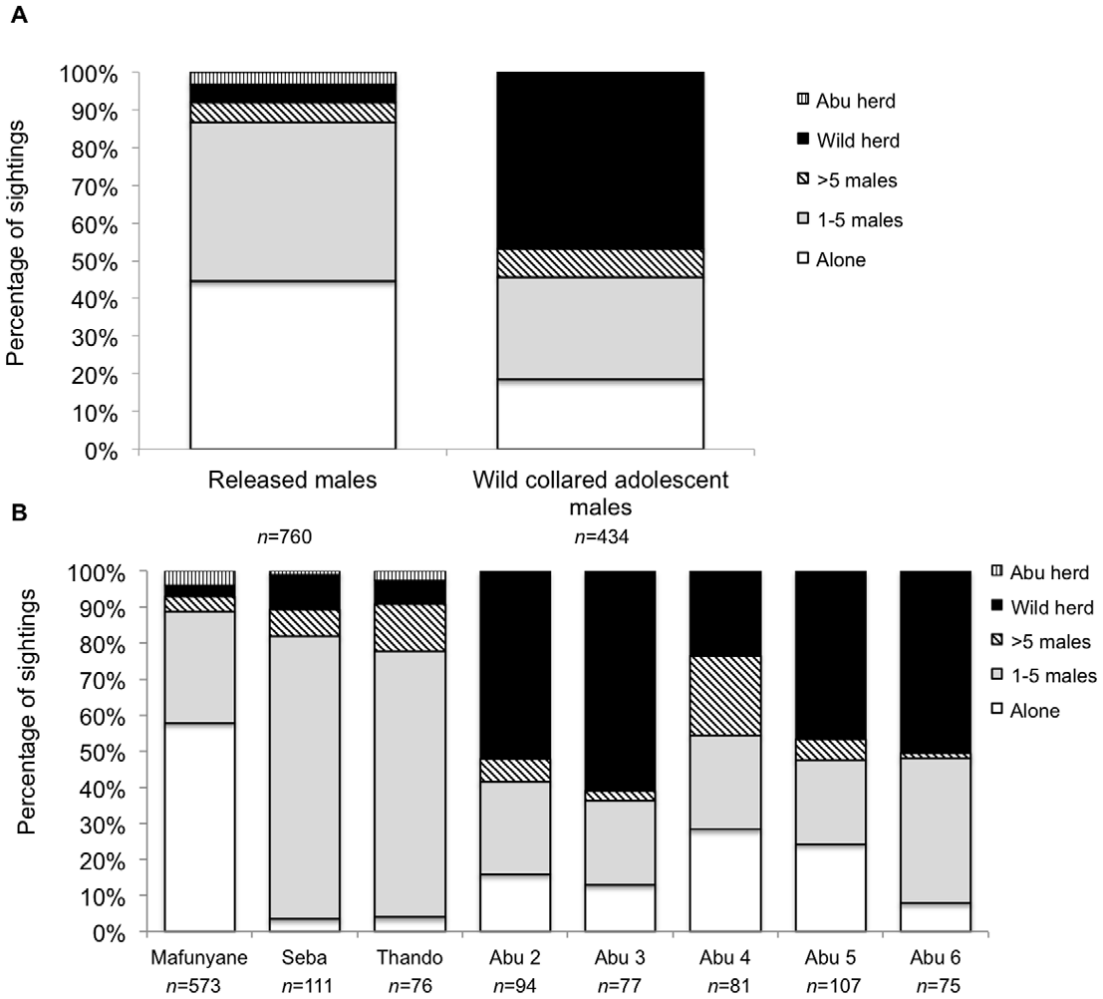
Percentage of sightings for which the released and wild collared adolescent male elephants were in different social groupings (Table 2). (a) total for the released and wild collared adolescent male elephants, and (b) for Mafunyane, Seba and Thando and wild collared adolescent males. *n* = number of independent sightings.

Prediction (ii), that the released males would tend to be alone or in smaller groups, was supported. A saturated log-linear model run in R showed that the released and wild collared males were significantly different in social groupings (Δdeviance_1_ = 285.21, P<0.001). Released males were seen alone significantly more often whereas the wild collared males spent more time with herds (Figure 1a,b), but the number of males seen with the released males did not differ significantly from the wild adolescent males (Mann-Whitney: W_3, 5_ = 8.0, P = 0.136). Of the released males, Mafunyane spent the majority of his time alone and differed from wild collared males in the time spent in different social groupings (Figure 1a,b; χ^2^_3_ = 696, P<0.001). With increasing time post release, he spent more time with other males and herds (Kruskal-Wallis H_2_ = 7.31, P = 0.026). Seba and Thando were seen most frequently with each other (Figure 1b); this differed significantly from wild collared males (Seba: χ^2^_3_ = 152, P<0.001; Thando: χ^2^_3_ = 95, P<0.001). Seba showed a significant difference in the number of sightings in particular social groupings (Table 2) in the first and second year post release (W_102, 53_ = 4848.5, P = 0.023) as he spent more time away from Thando, whereas there was no significant change for Thando (W_89, 56_ = 6259.5, P = 0.250).

**Figure 2:**
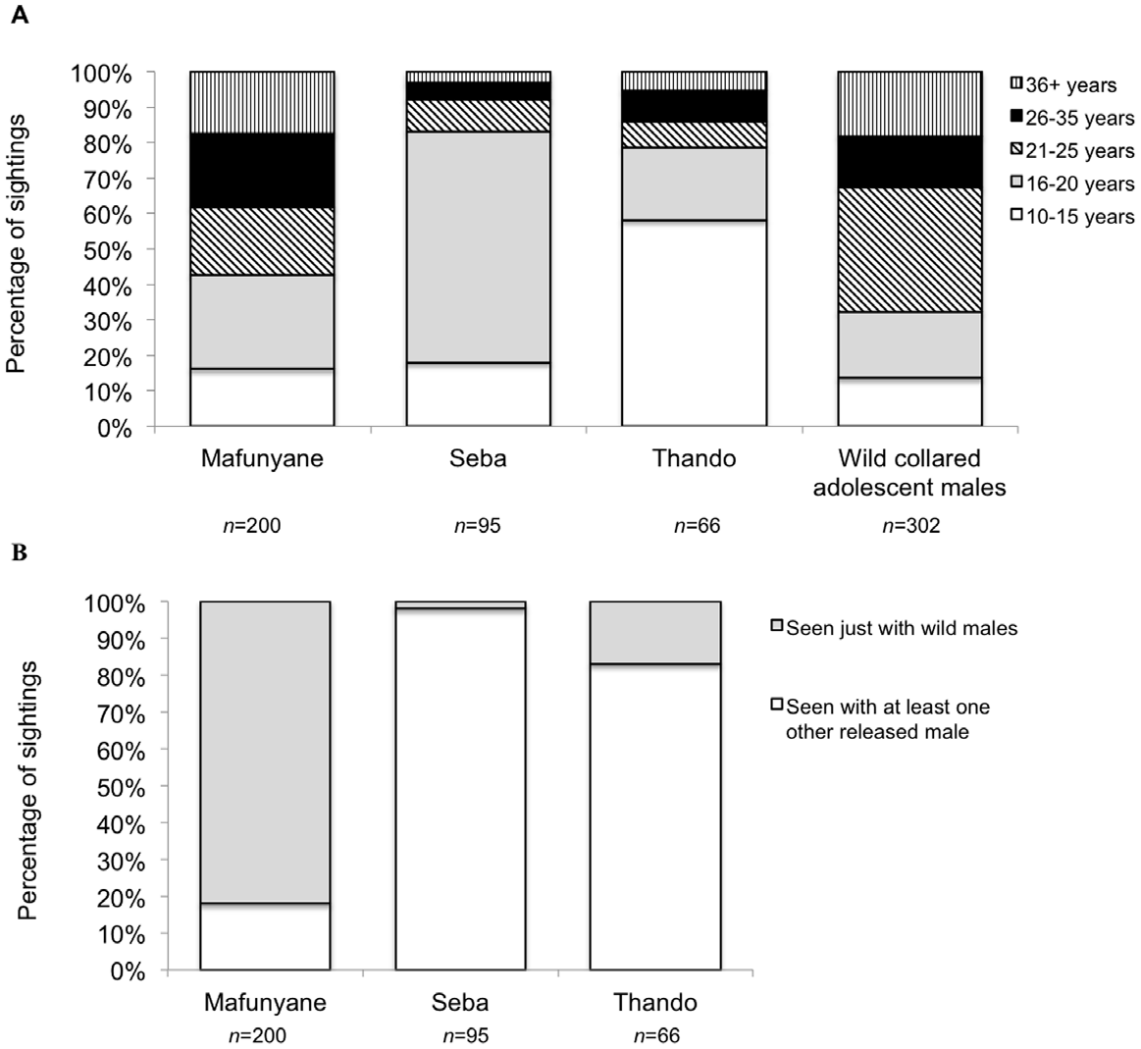
Percentage of sightings for which the released and wild collared adolescent male elephants were with males of different age groups. (a) all sightings with other male elephants, and (b) percentage of sightings of the released males with wild males. *n* = number of independent sightings.

**Figure 3:**
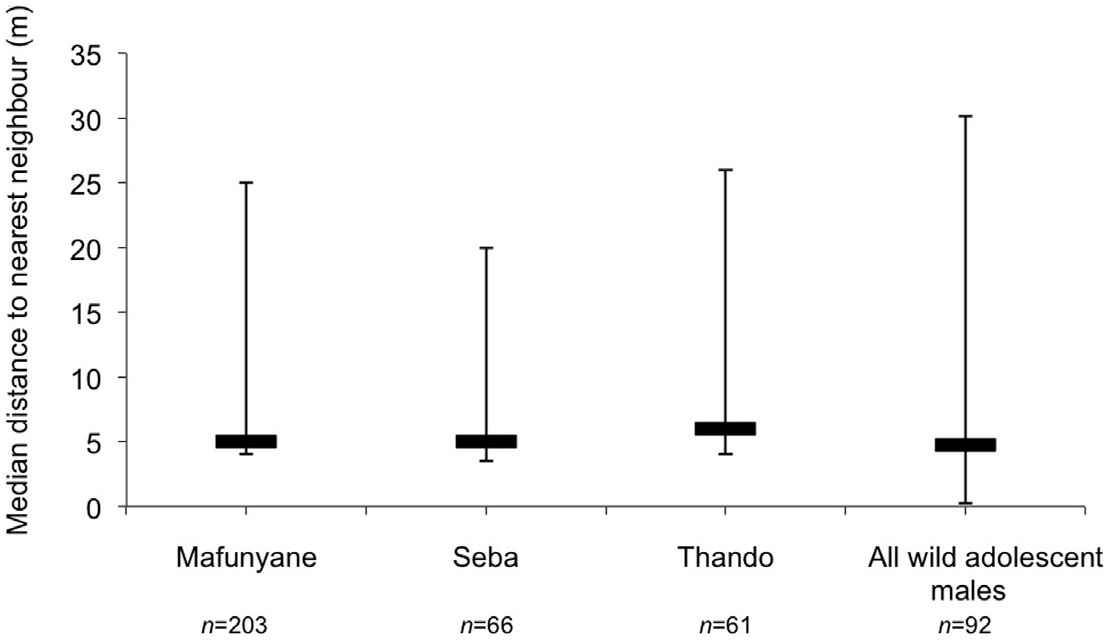
Median (±95% confidence limits) distance (m) of the focal elephant from its nearest neighbour averaged across each half-hour focal period. The wild sample included both collared and uncollared adolescent males. *n = *number of focal samples.

**Figure 4:**
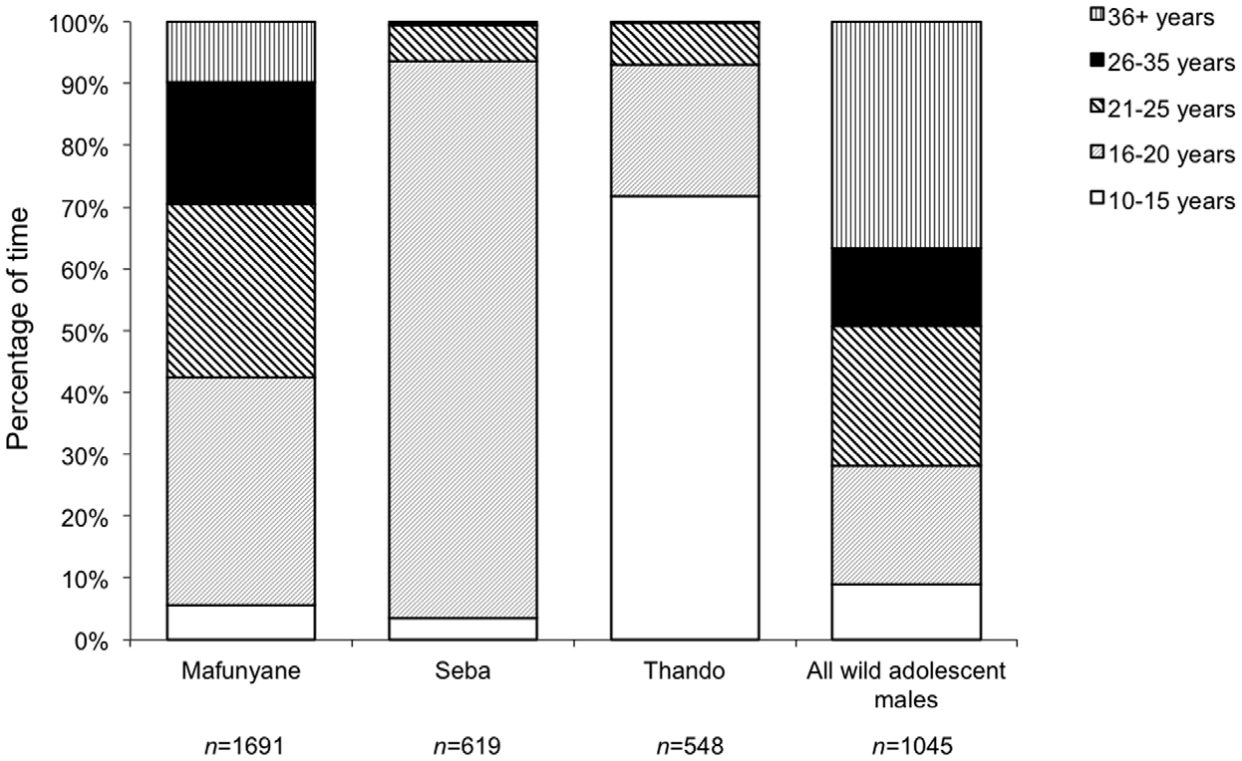
Percentage of time that the released and wild adolescent male elephants spent with males of different age classes as their nearest neighbour. The wild sample included both collared and uncollared adolescent males. *n* = total number of observations of nearest neighbour during those focal periods where another male was present.

The released males were seen more frequently with adolescent than older males (Mafunyane: χ^2^_4_ = 49.87, *P*<0.001; Seba: χ^2^_4_ = 455.63 *P*<0.001; Thando: χ^2^_4_ = 108.84, *P*<0.001; all released males: χ^2^_4_ = 43.78, *P*<0.001). A saturated log-linear model run in R showed a significant difference between the age groups with which the released and wild adolescent males associated (Figure 2: Δdeviance_4_ = 87.77, *P* = <0.001) but not the number of males (*W*_113, 388_ = 28813.5, *P* = 0.722). Distance to nearest neighbour did not differ significantly between the released and wild adolescent males (Figure 3: *W*_3, 101_ = 5249, *P* = 0.303). However, the released males spent more time closer to other adolescent males (Figure 4), whereas wild adolescent males spent more time closer to older males (10–15 years, χ^2^_3_ = 8.65, *P* = 0.03; 16–20 years, χ^2^_4_ = 70.94, *P*<0.001).

**Figure 5:**
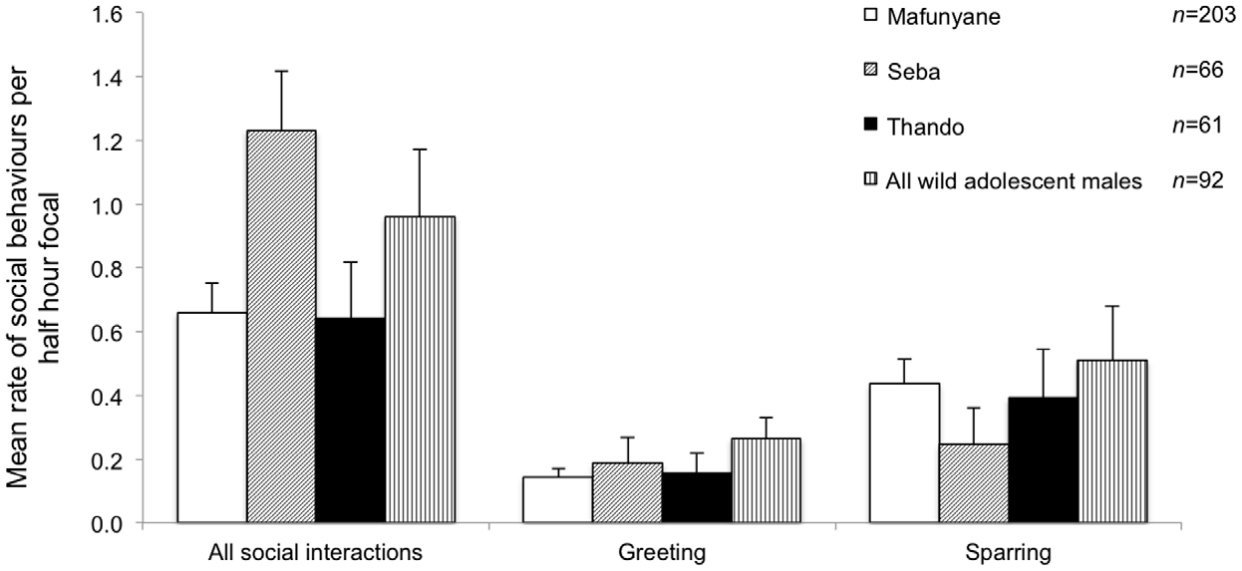
Mean (+SE) number of all social interactions, greeting and sparring per half-hour focal of the released and wild adolescent male elephants. The wild sample included both collared and uncollared adolescent males. *n = *number of focal periods when other elephants (both males and females) were present.

**Figure 6:**
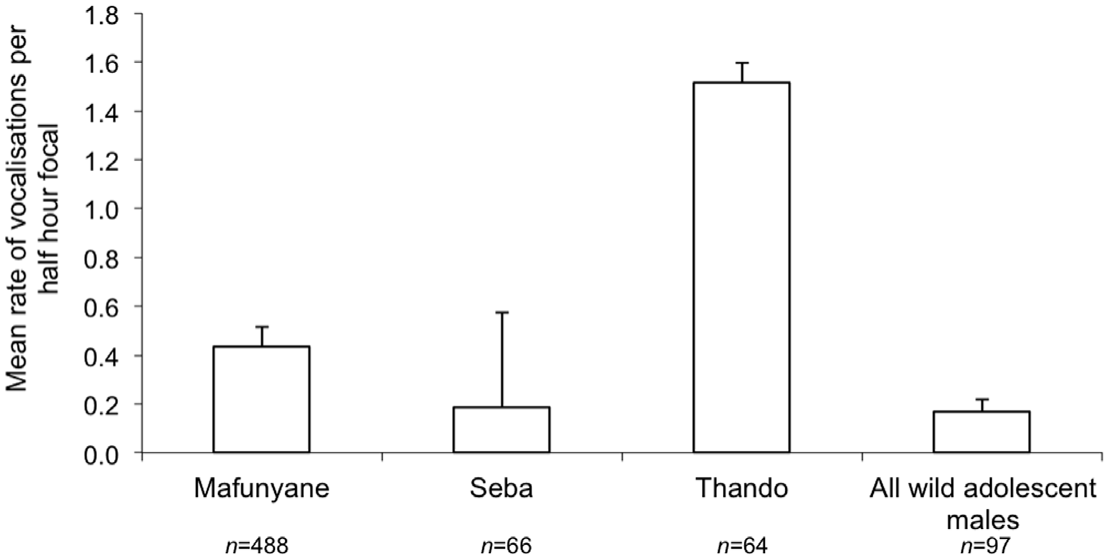
Mean (+SE) number of vocalisations per half-hour focal of the released and wild adolescent male elephants. The wild sample included both collared and uncollared adolescent males. *n = *number of focal periods.

Contrary to prediction (iii), there was no significant difference between the total number of social interactions of the released and wild adolescent males (Figure 5: F_1, 430_ = 0.02, P = 0.899) or between the three released males when considered separately (F_2, 327_ = 1.77, P = 0.171). There was no significant difference between the number of greetings per half hour for the released and wild males (Figure 5: Poisson χ^2^_430_, P = 0.254) but there was between the three released males (Poisson χ^2^_327_, P = 0.029), with Seba having the highest mean value. Wild adolescent males sparred significantly more per half hour than the released males (Figure 5: F_1, 430_ = 7.075, P = 0.008). There was also a significant difference between the released males (F_2, 327_ = 3.399, P = 0.035), with Mafunyane having the highest mean value. Released and wild males differed significantly in the rate of vocalisation (Figure 6: F_1, 718_ = 14.425, P<0.001), with released males vocalising more often. There was also a significant difference between the released males (F_2, 615_ = 4.66, P = 0.010): Thando was more likely to vocalise, but when Mafunyane vocalised, he did so more often during a half-hour focal.

Contrary to prediction (iv), mean±SE 100% MCP (Table 5) of the released males (2202±372 km^2^) was not significantly different from the wild males (4824±1431 km^2^; t_4_ = −1.51, P = 0.206). Mean±SE 100% MCP of the released males was 2035±382 km^2^ in the rainy season, 1322±48 km^2^ in the flood season; neither were significantly different from the wild males (3837±2540 km^2^; t_4_ = −0.01, P = 0.996; 2479±689 km^2^; t_4_ = −2.25, P = 0.088, respectively). However, the released males had significantly smaller core areas (mean±SE 70% kernel; 254±53 km^2^) than the wild males (787±372 km^2^; t_4_ = −3.15, P = 0.034) during the rainy but not the flood season (released males 162±43 km^2^; wild males 269±72 km^2^; t_4_ = −1.28, P = 0.258).

**Table 5:** Area used (100% MCPs and 95% kernels) and core area (70% kernels) for the released and wild collared adolescent male elephants.

Elephant	100% MCP(km^2^)	95% kernel(km^2^)	70% kernel(km^2^)	Date of first fix used	Date of last fix used	Number of GPS locations used
Mafunyane	2003	653	282	1 Feb 2002	1 Feb 2005	1464
Seba	1679	992	285	10 Feb 2003	1 Feb 2005	1788
Thando	2924	1317	285	10 Feb 2003	1 Feb 2005	1674
Abu2	4551	1657	391	16 Feb 2002	1 Feb 2005	1825
Abu3	4450	1094	256	17 Feb 2002	14 July 2004	1617
Abu4	3228	2440	807	16 Feb 2002	9 Nov 2004	2160
Abu5	1721	600	118	16 Feb 2002	1 Feb 2005	2678
Abu6	10,168	3183	820	17 Feb 2002	1 Feb 2005	2521

**Figure 7:**
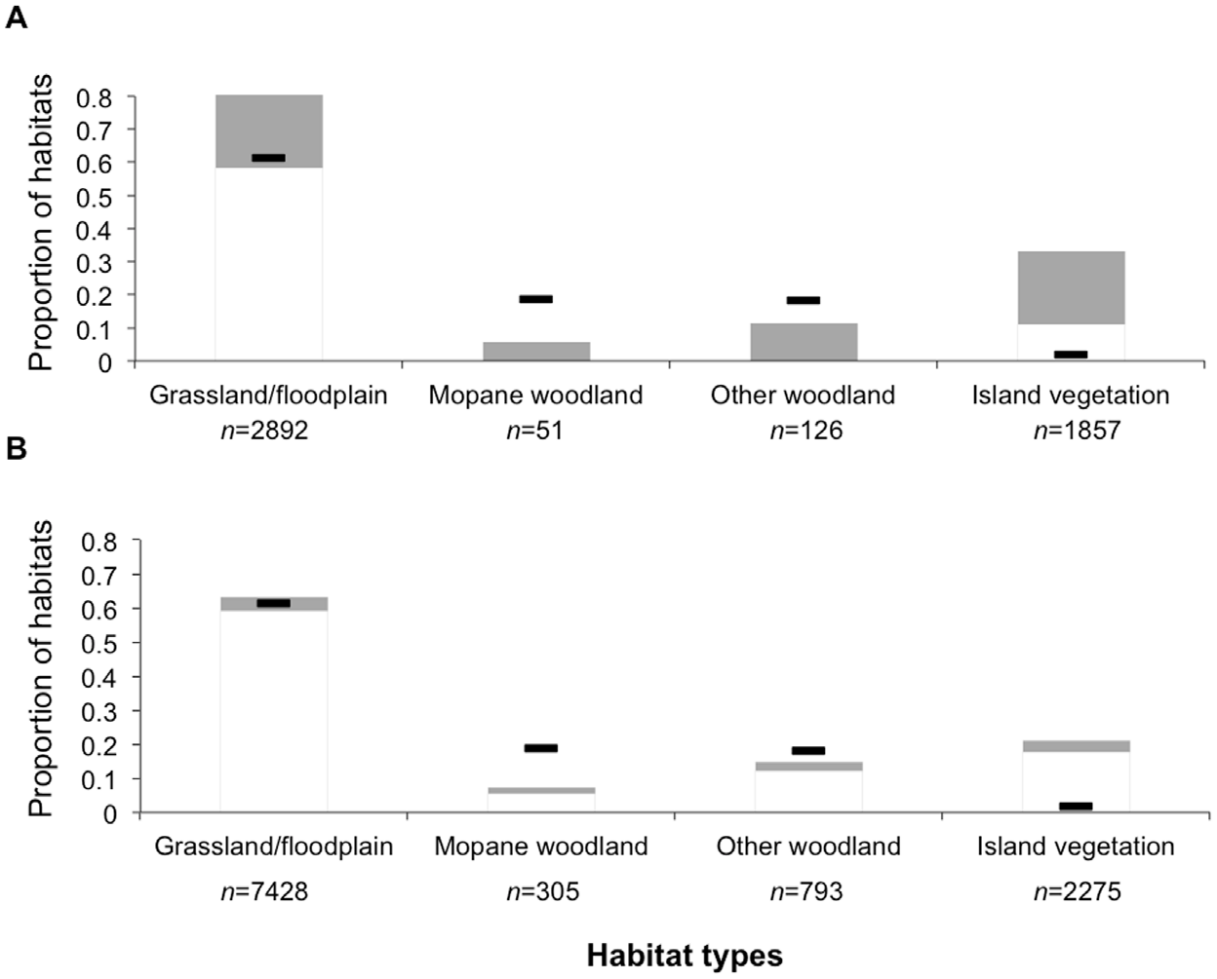
Bonferroni-adjusted confidence limits for the proportion of each habitat used (shaded boxes) compared to availability (dark lines) for all seasons combined. (a) released males, and (b) wild collared adolescent males. *n* = number of GPS locations.

Prediction (v) was not supported. Habitat use was similar and non-random for both released and wild males (Figure 7a,b: log-likelihood χ^2^: χ^2^_3_ = 6109, *P*<0.001; χ^2^_5_ = 6109, *P*<0.001, respectively), although wild males used grassland/floodplain more than the released males. There was no difference between released and wild males when their 100% MCPs were compared to the total available habitat (*F*_1, 23_ = 0.31, *P* = 0.587) or when the habitat used was compared to that available in their 100% MCPs (*F*_1, 23_ = 1.56, *P* = 0.226).

## Discussion

There are nearly 700 translocation programmes annually in the USA alone [2], [11], and worldwide the number is expected to rise considerably as wild populations become increasingly fragmented and/or endangered, and in attempts to restore dysfunctional ecosystems [46], [47]. For some species, captive breeding and release is the only means of survival in the wild [48], [49]. However, the ability of released animals to integrate into wild populations varies between species and with age at release, amount of contact with humans prior to release, release procedures, and release site characteristics [11], [15], [18], [50].

While survival long enough to breed is widely used as a measure of release success [51], musth in male elephants does not start until they are in their late twenties or early thirties [26], and males generally do not breed for several more years. So we used other measures of success. While the released males did not associate with humans or pose a risk to human safety, it was not surprising that initially all three released males spent time close to, and at times in vocal communication with, the Abu herd. The transition to independence in African elephants is gradual, normally taking one to four years, but can take longer [52], [53], and so the separation experienced by the three released males was abrupt.

After the intensive monitoring period, the released males slowly expanded the area they explored and spent more time with wild elephants. In 2006, Seba left the protected area of the Okavango Delta through a fence flattened by floodwaters, and in December 2010, 94 months after release, he and 10 other elephants were shot 140 km from the release site on a ranch where elephants had been damaging cattle fences. The affected farmers said this was generally done by females: while males usually step over the low fences, females break them so their calves can get through. They confirmed that Seba was not acting any differently from the wild elephants; he was shot in mistake for a female. From 2006 Seba’s average distance from human habitation was the greatest of the released males, and there is no evidence that he was attracted to the farming area because of human presence. With 70% of African elephants currently living outside protected areas [54], we believe this event was a reflection of the current pattern of human-elephant conflict, not because Seba failed to integrate into the wild. Typically, only a minority of wild-born male elephants survive to the end of adolescence [55]. Seba survived in the wild for 94 months and was reaching the end of adolescence. Mafunyane and Thando had survived 115 and 103 months when their collars were removed, and Thando had experienced his first musth.

The ability of captive-raised animals to integrate into a complex social system such as that of male African elephants is a key indicator of welfare and release success [56]. In the early years the released males were not fully integrated into wild bull society. Mafunyane spent the majority of his time alone, and Seba and Thando were together 80% of the time. Adolescent male elephants often leave their natal herd together and socialise with each other more than other males [57], as do many mammals [58], [59], and so releasing adolescent male elephants in groups may aid their integration into the wild [4], [60]. It may also explain why the released males, unlike the wild males, spent more time closer to other adolescent males. The wild males would have become familiar with many of the other young males prior to leaving their natal herds, and so could invest more time getting to know the older bulls. Although patterns of social behaviour differed from wild adolescent males in some respects, the released males were integrating into the fission-fusion society of the wild elephants, and interactions between the released and wild elephants were non-aggressive.

Neither range area nor habitat use differed significantly between the released and wild males. Although Mafunyane spent the majority of his time in a very small part of his range, Seba and Thando were more exploratory, perhaps again reflecting the advantages of releasing small groups of adolescent males. After the intensive monitoring period, the released males continued to expand their ranges and spent more time with wild elephants, interacting with both herds and bulls. So the lack of time spent with older bulls following their release did not appear to be a significant issue in the long term.

### Conclusions

African elephants have disappeared from many of their range states [54], and there are likely to be significant conservation and ecological benefits from restoring flagship species to areas they cannot recolonise naturally [61]. However, it is important to release the right age mixture and sex ratio [15], [62]. Despite generally low success rates with captive-raised animals, our data show that it is possible to release captive-raised male elephants without significant welfare concerns, that survival rates post-release can be high, and that they can integrate into the complex society of bull elephants. Releasing captive-raised elephants is controversial and only an option where there are established elephant populations, since the presence of older bulls is a prerequisite for a stable bull society [63]. While time-consuming and expensive, it is almost certainly cheaper than keeping unwanted elephants in captivity. Our data suggest this should be explored further.
